# Association between Oral Streptococcus Mutans Counts and Proteinuria in Patients with Chronic Kidney Disease: A Pilot Study Using Chlorhexidine

**DOI:** 10.31662/jmaj.2025-0375

**Published:** 2025-11-21

**Authors:** Taro Misaki, Yuto Suehiro, Shuhei Naka, Daiki Matsuoka, Kana Suehara, Seigo Ito, Yasuyuki Nagasawa, Rena Okawa, Ryota Nomura, Michiyo Matsumoto-Nakano, Kazuhiko Nakano

**Affiliations:** 1 Division of Nephrology, Seirei Hamamatsu General Hospital, Hamamatsu, Shizuoka, Japan; 2 Department of Nursing, Faculty of Nursing, Seirei Christopher University, Hamamatsu, Shizuoka, Japan; 3 Joint Research Laboratory of Science for Oral and Systemic Connection, Graduate School of Dentistry, The University of Osaka, Suita, Osaka, Japan; 4 Department of Pediatric Dentistry, Graduate School of Dentistry, The University of Osaka, Suita, Osaka, Japan; 5 Department of Pediatric Dentistry, Okayama University Graduate School of Medicine, Dentistry and Pharmaceutical Sciences, Okayama, Okayama, Japan; 6 Department of Internal Medicine, Japan Self-Defense Force Iruma Hospital, Iruma, Saitama, Japan; 7 Department of General Internal Medicine, Hyogo Medical University, Nishinomiya, Hyogo, Japan; 8 Department of Pediatric Dentistry, Graduate School of Biomedical and Health Sciences, Hiroshima University, Hiroshima, Japan

**Keywords:** Oral care, chlorhexidine mouthwash, *Streptococcus mutans*, CKD, proteinuria

## Abstract

**Introduction:**

Chlorhexidine mouthwash is one of the most widely used anti-microbial agents, reducing oral cavity bacterial load. However, the effects of mouthwash on systemic conditions in patients with chronic kidney disease (CKD) remain unknown. We examined the relationship between *Streptococcus mutans* abundance in the oral cavity and proteinuria in patients with CKD.

**Methods:**

Patients with CKD (n = 57) gargled with mouthwash containing chlorhexidine gluconate three times daily for 1 year. We prospectively investigated the relationship between changes in the number of *S. mutans* and proteinuria.

**Results:**

The number of *S. mutans* colony-forming units (CFU) ≥10^3^/mL saliva at month 0 was significantly associated with higher urinary proteinuria and *S. mutans* CFU/mL over time. The mean number of *S. mutans* in all patients decreased significantly after 12 months. Proteinuria also decreased significantly after 12 months compared with after 6 months. The rate of proteinuria <0.3 g/g creatinine after 12 months was significantly higher in the group that had <10^3^ CFU/mL *S. mutans* after 12 months than in other groups.

**Conclusions:**

In this pilot study, a potential association was observed between oral *S. mutans* counts and proteinuria in patients with CKD. Larger studies are needed to clarify this relationship.

## Introduction

Dental caries and periodontitis are two major dental diseases. *Streptococcus mutans*, a Gram-positive facultative anaerobic bacterial species found in supragingival locations, is a major pathogen in dental caries ^(1)^. Several bacterial species, mainly Gram-negative obligate anaerobes found in subgingival areas, are considered to be involved in periodontitis pathogenesis ^(2)^.

To prevent these dental diseases, regular check-ups and cleanings by dental professionals and daily self-care are important ^(3), (4)^. Among the various self-care products available, mouthwash is one of the easiest to use for those who want to improve their oral hygiene ^(5)^. Chlorhexidine mouthwash is one of the most widely used anti-microbial agents by oral health care practitioners and individuals with and without oral disease; it reduces bacterial load within the oral cavity to prevent and manage oral disease ^(6), (7)^. Chlorhexidine mouthwash is known to have anti-microbial effects on major oral pathogens such as *S. mutans,* as well as several periodontitis-related bacterial species ^(8)^. However, the effects of mouthwash on systemic conditions in patients with chronic kidney disease (CKD) remain unknown.

Recently, we demonstrated that several oral bacteria are associated with immunoglobulin A (IgA) nephropathy (IgAN), one of the causes of CKD ^(9), (10), (11), (12), (13), (14), (15), (16), (17), (18), (19), (20), (21)^, including *S. mutans* ^(10), (11), (12), (13), (14), (15), (17), (19), (21)^. In this exploratory study, we aimed to investigate whether the use of chlorhexidine-containing mouthwash could reduce *S. mutans* oral abundance and whether such changes may be associated with proteinuria improvements in patients with CKD.

## Materials and Methods

### Patients and clinical characteristics

The subjects were patients with CKD (n = 57) who were outpatients of Seirei Hamamatsu General Hospital in Hamamatsu, Japan. In this study, CKD was defined as including IgAN, chronic glomerulonephritis, diabetic nephropathy, nephrosclerosis, and others. Primary diseases in patients with CKD included IgAN (n = 10), chronic glomerulonephritis (n = 9), diabetic nephropathy (n = 19), nephrosclerosis (n = 17), and others (n = 2). Patients currently taking immunosuppressive drugs were excluded, and no immunosuppressive drugs were used during the study period. All eligible patients were at least 20 years old, and consent forms were obtained from each patient.

### Gargling with mouthwash

The patients were instructed to gargle with 25 mL of water with mouthwash for 30 seconds three times a day (morning, afternoon, and night) for 12 months. The mouthwash used in this study was ConCool F^Ⓡ^ (Weltec Corp., Osaka, Japan), which contains 0.05% chlorhexidine gluconate. When gargling with mouthwash, patients were asked to dilute it to approximately 0.00056% (10 drops in 25 mL tap water for each gargle) ^(8)^. A gargling logbook was provided to each participant to document daily mouthwash use; however, as many patients did not return the logbook, adherence data could not be included in the statistical analysis.

### Data collection

Saliva specimens were obtained from patients every 2-3 months. Patients were followed prospectively for 1 year from May 2022 to October 2023 (registration number UMIN000051221). Non-stimulated expectorated whole saliva was collected from each subject in a sterile plastic tube and stored at −20°C. Clinical data (height, body weight, body mass index, systolic blood pressure, diastolic blood pressure, hemoglobin, serum albumin, serum creatinine, estimated glomerular filtration rate [eGFR], urinary protein excretion (g/g creatinine; g/gCr), percentage of urinary protein ≥2+, percentage of urinary sediment ≥5 red blood cells (RBC)/high power field (HPF) or higher, and renin-angiotensin system inhibitor medication rate) from patients with CKD were evaluated at the time of saliva specimen collection. Fifty-one out of 57 patients were able to be followed up for 1 year.

### Analysis of *S. mutans*

Frozen saliva specimens were used to count the number of* S. mutans*, as described previously with slight modifications ^(11)^. In brief, 1 mL saliva specimens were diluted and streaked onto Mitis-Salivarius agar plates (Difco Laboratories, Detroit, MI, USA) containing bacitracin (0.2 U/mL; Sigma-Aldrich, St. Louis, MO, USA) and 15% (wt/vol) sucrose. After anaerobic incubation at 37°C for 48 hours, the number of colonies were counted to determine the number of *S. mutans* present ^(11)^.

### Statistical analysis

**Figure 1.**
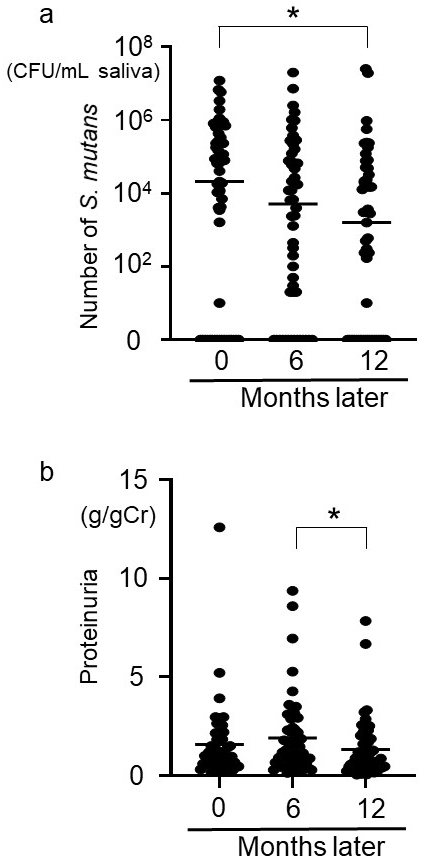
Changes over time and relationship between the number of* S. mutans* and proteinuria. Change in the number of *S. mutans* over time (a)*.* Change in proteinuria over time (b). p < 0.05 was considered statistically significant in the Friedman test, and p < 0.0167 was considered statistically significant in the Bonferroni correction (*). *S. mutans*:* Streptococcus mutans*.

**Figure 2.**
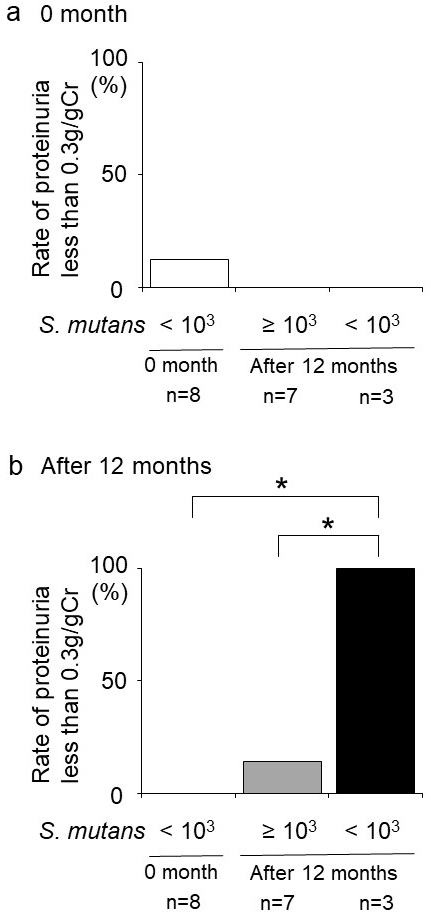
Relationship between reaching *S. mutans* <10^3^ CFU/mL and achieving proteinuria <0.3 g/gCr after 12 months in patients with IgA nephropathy or chronic glomerulonephritis. (a) Rate of proteinuria <0.3 g/gCr at month 0 in patients with IgA nephropathy or chronic glomerulonephritis. (b) Rate of proteinuria <0.3 g/gCr after 12 months in patients with IgA nephropathy or chronic glomerulonephritis. p < 0.0167 was considered statistically significant in the Bonferroni correction (*). CFU: colony-forming unit; *S. mutans*:* Streptococcus mutans*.

All results are expressed as mean ± standard deviation (SD). When there was a significant difference, a further statistical analysis was conducted using Fisher’s protected least significant difference test or Fisher’s exact test between two groups. For comparisons between two groups that were not normally distributed, we performed Mann-Whitney U tests (urinary protein (g/gCr; mean ± SD), number of *S. mutans*) (Table 1). The Friedman test and Bonferroni correction were used in repeated measures analysis (Figure 1). Logistic regression analysis was used for correlation analysis (Table 2 and 3). Bonferroni correction was also used in Table 4 and Figure 2. Proteinuria over time was evaluated by comparing the ≥ and <10^3^ CFU/mL groups using a mixed-effects model. In these analyses, *P* < 0.05 was considered statistically significant. *P* < 0.0167 was considered statistically significant in the Bonferroni correction. Statistical analyses were conducted using Statview (SAS Institute Inc., Cary, NC, USA), GraphPad Prism 8 (San Diego, CA, USA), and JMP software (version 14; SAS Institute Inc., Cary, NC, USA).

**Table 1.** Relationship between *S. mutans* Abundance and Proteinuria.

Characteristics	*S. mutans* <10^3^ CFU/mL	*S. mutans* ≥10^3^ CFU/mL	p-Value
**0 month**	(n = 18)	(n = 39)	
**Age (yr; mean ± SD)**	**60.8 ± 18.1**	**69.2 ± 12.7**	**0.0478**
Sex (M/F)	12/6	29/10	0.5563
Height (cm; mean ± SD)	165.1 ± 12.2	163.6 ± 7.9	0.5907
Body weight (kg; mean ± SD)	63.7 ± 17.9	63.9 ± 15.7	0.9628
BMI (kg/m^2^; mean ± SD)	23.0 ± 4.1	23.7 ± 4.9	0.5871
Systolic blood pressure (mmHg; mean ± SD)	127.7 ± 15.4	136.7 ± 19.2	0.0843
Diastolic blood pressure (mmHg; mean ± SD)	75.0 ± 10.7	76.7 ± 13.8	0.6431
**Hemoglobin (g/dL; mean ± SD)**	**13.0 ± 2.6**	**11.5 ± 1.8**	**0.0151**
**Serum albumin (g/dL; mean ± SD)**	**4.0 ± 0.5**	**3.7 ± 0.3**	**0.032**
Serum creatinine (mg/dL; mean ± SD)	2.0 ± 1.4	2.3 ± 1.2	0.3303
eGFR (mL/min/1.73 m^2^; mean ± SD)	38.1 ± 21.3	29.6 ± 20.8	0.1619
Urinary protein (g/gCr; mean ± SD)	1.0 ± 0.8	1.9 ± 2.1	0.1170
**% Urinary protein ≥2+**	**33.3**	**66.7**	**0.018**
**Percentage of urinary sediment ≥5 RBC/HPF**	**0**	**23.1**	**0.0263**
RAS-I medication rate, %	66.7	59.0	0.5872
**6 months later**	(n = 18)	(n = 38)	
**Number of *S. mutans* (log)**	**0.5 ± 0.8**	**4.4 ± 1.5**	**<0.0001**
eGFR (mL/min/1.73 m^2^; mean ± SD)	38.9 ± 21.1	29.0 ± 20.6	0.1011
**Urinary protein (g/gCr; mean ± SD)**	**1.0 ± 0.6**	**2.3 ± 2.2**	**0.0134**
**% Urinary protein ≥2+**	**38.9**	**46.8**	**0.0388**
Percentage of urinary sediment ≥5 RBC/HPF	11.1	23.7	0.277
**12 months later**	(n = 18)	(n = 33)	
**Number of *S. mutans* (log)**	**0.2 ± 0.7**	**4.1 ± 1.6**	**<0.0001**
eGFR (mL/min/1.73 m^2^; mean ± SD)	36.8 ± 21.4	27.5 ± 20.6	0.1251
**Urinary protein (g/gCr; mean ± SD)**	**0.8 ± 0.7**	**1.7 ± 1.7**	**0.0162**
**% Urinary protein ≥2+**	**27.8**	**67.6**	**0.0048**
Percentage of urinary sediment ≥5 RBC/HPF	11.1	16.2	0.6221

Bold values indicate statistical significance at p < 0.05. BMI, body mass index; CFU: colony forming unit; eGFR, estimated glomerular filtration rate; g/gCr: g/g creatinine; HPF: high power field; RAS-I, renin-angiotensin system inhibitor; RBC: red blood cell; *S. mutans*: *Streptococcus mutans*; SD: standard deviation.

**Table 2.** Relationship between *S. mutans* ≥10^3^ CFU/mL and % Urinary Protein ≥2+ at Month 0.

Variables	Odds ratio (95% confidence interval)	p-Value
Age	1.010 (0.955-1.067)	0.7323
Sex	3.949 (0.821-18.994)	0.0865
**Systolic blood pressure (mmHg)**	**1.047 (1.003-1.093)**	**0.0346**
eGFR (mL/min/1.73 m^2^)	0.985 (0.948-1.024)	0.4519
Hemoglobin (g/dL)	1.246 (0.823-1.885)	0.2982
RAS-I medication	1.852 (0.452-7.578)	0.3915
***S. mutans* ≥10^3^ CFU**/mL	**4.679 (1.008-21.732)**	**0.0489**

Logistic regression models adjusted for age, sex, systolic blood pressure, eGFR, hemoglobin, and RAS-I medication were used. The association between the *S. mutans* ≥10^3^ CFU/mL and % urinary protein ≥2+ remained statistically significant in subsequent logistic regression analysis. Independent variables were age, sex, systolic blood pressure, eGFR, hemoglobin, RAS-I medication, and* S. mutans* ≥10^3^ CFU/mL. The dependent variable was % urinary protein ≥2+. Bold value indicates statistical significance at p < 0.05. CFU: colony forming unit; eGFR, estimated glomerular filtration rate; RAS-I, renin-angiotensin system inhibitor;* S. mutans*:* Streptococcus mutans*.

**Table 3.** Relationship between the Degree of Decrease in the Number of *S. mutans* and Proteinuria <0.3 g/gCr after 12 Months.

Variables	Odds ratio (95% confidence interval)	p-Value
Age	0.934 (0.799-1.091)	0.3873
Sex	1.442 (0.050-42.000)	0.8314
BMI (kg/m^2^)	0.723 (0.427-1.226)	0.2292
Systolic blood pressure (mmHg)	1.031 (0.877-1.213)	0.7092
eGFR (mL/min/1.73 m^2^)	1.097 (0.98-1.227)	0.1076
Proteinuria (g/gCr) (month 0)	0.028 (0.001-1.414)	0.0740
RAS-I medication	9.834 (0.082-1179.978)	0.3493
**Degree of decrease in the number of *S. mutans* after 12 months**	**0.022 (0.001-0.945)**	**0.0466**

Logistic regression models adjusted for age, sex, BMI, systolic blood pressure, eGFR, proteinuria at month 0, and RAS-I medication were used. The association between the decrease in the number of *S. mutans* and proteinuria <0.3 g/gCr after 12 months remained statistically significant in subsequent logistic regression analysis. Independent variables were age, sex, BMI, systolic blood pressure, eGFR, proteinuria at month 0, RAS-I medication, and the degree of decrease in the number of *S. mutans* after 12 months. The dependent variable was proteinuria <0.3 g/gCr after 12 months. Bold value indicates statistical significance at p < 0.05. BMI, body mass index; eGFR, estimated glomerular filtration rate; g/gCr: g/g creatinine; RAS-I, renin-angiotensin system inhibitor; *S. mutans*:* Streptococcus mutans*.

**Table 4.** Relationship between Reaching *S. mutans* <10^3^ CFU/mL and Achieving Proteinuria <0.3 g/gCr after 12 Months.

Characteristics	*S. mutans* <10^3^ CFU/mL at month 0 (n = 18)	P-value	*S. mutans* ≥10^3^ CFU/mL at 12 months (n = 27)	P-value	*S. mutans* <10^3^ CFU/mL at 12 months (n = 6)
**Chronic kidney disease subtypes**					
Rate of IgA nephropathy, %	27.8	0.7555	7.4	0.1334	33.3
Rate of chronic glomerulonephritis, %	16.7	0.9999	18.5	0.9173	16.7
Rate of nephrosclerosis, %	33.3	0.4461	22.2	0.1874	50.0
Rate of diabetic nephropathy, %	22.2	0.3033	48.1	0.0227	0
**Month 0**					
Age (yr; mean ± SD)	60.8 ± 18.1	0.8411	68.1 ± 11.8	0.3721	62.2 ± 14.5
Sex (M/F)	12/6	0.9999	19/8	0.8644	4/2
**Number of *S. mutans* (log)**	**0.1 ± 0.2**	**<0.0001**	**5.2 ± 0.9**	**0.0014**	**4.2 ± 0.8**
BMI (kg/m^2^; mean ± SD)	23.0 ± 4.1	0.1952	23.6 ± 4.7	0.2825	25.9 ± 7.0
Systolic blood pressure (mmHg; mean ± SD)	165.1 ± 12.2	0.7015	127.7 ± 15.4	0.2365	136.7 ± 19.2
Diastolic blood pressure (mmHg; mean ± SD)	75.0 ± 10.7	0.9343	77.8 ± 14.0	0.6949	75.5 ± 13.0
Hemoglobin (g/dL; mean ± SD)	13.0 ± 2.6	0.3044	11.8 ± 1.7	0.7991	12.0 ± 1.9
Serum albumin (g/dL; mean ± SD)	4.0 ± 0.5	0.3623	3.7 ± 0.4	0.6964	3.8 ± 0.3
Serum creatinine (mg/dL; mean ± SD)	2.0 ± 1.4	0.9008	2.2 ± 1.1	0.5669	1.9 ± 1.1
eGFR (mL/min/1.73 m^2^; mean ± SD)	38.1 ± 21.3	0.8802	30.8 ± 22.6	0.5632	36.5 ± 18.9
Urinary protein (g/gCr; mean ± SD)	1.0 ± 0.8	0.6453	2.0 ± 2.4	0.5299	1.5 ± 1.2
Rate of proteinuria <0.3 g/gCr (%)	5.6	0.5572	3.7	0.6824	0
**Percentage of urinary sediment** ≥5 **RBC/HPF**	**0**	**0.0017**	14.8	0.0185	**50.0**
RAS-I medication rate, %	66.7	0.4736	55.6	0.2147	83.3
**Month 12**					
**Number of *S. mutans* (log)**	**0.2 ± 0.7**	**0.0002**	**4.6 ± 1.2**	**<0.0001**	**2.0 ± 1.0**
eGFR (mL/min/1.73 m^2^; mean ± SD)	36.8 ± 21.4	0.6688	28.6 ± 21.9	0.6898	32.5 ± 17.8
Urinary protein (g/gCr; mean ± SD)	0.8 ± 0.7	0.9268	1.6 ± 1.5	0.1988	0.8 ± 1.0
**Rate of proteinuria <0.3 g/gCr (%)**	**5.6**	**0.0028**	**7.4**	**0.0028**	**50.0**
Percentage of urinary sediment ≥5 RBC/HPF	11.1	0.7525	18.5	0.9129	16.7

Bold values indicate statistical significance at p < 0.0167. p-Value: versus group of *S. mutans* <10^3^ CFU/mL at 12 months. BMI, body mass index; CFU: colony forming unit; eGFR, estimated glomerular filtration rate; g/gCr: g/g creatinine; HPF: high power field; RAS-I, renin-angiotensin system inhibitor; RBC: red blood cell; *S. mutans*:* Streptococcus mutans*; SD: standard deviation.

## Results

### The relationship between higher *S. mutans* oral cavity abundance and proteinuria in patients with CKD

**Figure 3.**
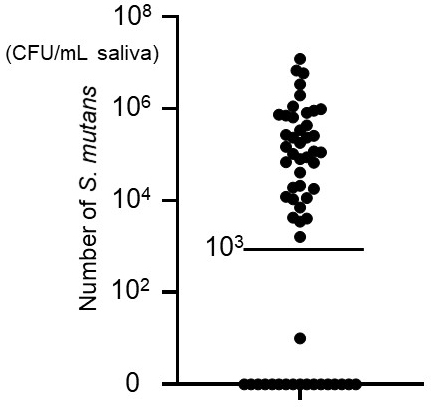
Number of *Streptococcus mutans* in saliva. The number of *S. mutans* in saliva from each patient at month 0.

Patients with < or ≥10^3^ CFU/mL *S. mutans* in the oral cavity at month 0 were divided into two groups (Figure 3) and compared over time (Table 1). No significant differences were found between the groups in terms of sex, height, body weight, body mass index, systolic blood pressure, diastolic blood pressure, serum creatinine, eGFR, urinary protein (g/gCr), and renin-angiotensin system inhibitor medication rate at month 0 (Table 1). The ≥10^3^ CFU/mL group was significantly associated with higher age, lower hemoglobin, lower serum albumin, higher percentage of urinary protein ≥2+, and higher percentage of urinary sediment ≥5 RBC/HPF at month 0 (Table 1). The relationship between *S. mutans* ≥10^3^ CFU/mL and urinary protein ≥2+ at month 0 remained significantly different in subsequent logistic regression analysis adjusted for age, sex, systolic blood pressure, eGFR, hemoglobin, and renin-angiotensin system inhibitor use (Table 2).

No significant difference was found between groups regarding eGFR and the percentage of urinary sediment ≥5 RBC/HPF at 6 months. The *S. mutans* ≥10^3^ CFU/mL group was significantly associated with a higher number of *S. mutans*, higher urinary protein (g/gCr), and a higher percentage of urinary protein ≥2+ at 6 months (Table 1). No significant difference was found between the two groups regarding eGFR and the percentage of urinary sediment ≥5 RBC/HPF after 12 months. The *S. mutans* ≥10^3^ CFU/mL group was significantly associated with a higher number of *S. mutans*, higher urinary protein (g/gCr), and a higher percentage of urinary protein ≥2+ after 12 months (Table 1). Proteinuria (g/gCr) at month 0 did not differ significantly between the ≥ and <10^3^ CFU/mL *S. mutans* groups. However, over time (0, 6, and 12 months), proteinuria was significantly associated with the ≥10^3^ CFU/mL group (mixed-effects model, adjusted for age, blood pressure, and eGFR; *P* = 0.0181).

### The relationship between decreases in oral cavity *S. mutans* and proteinuria in patients with CKD

**Figure 4.**
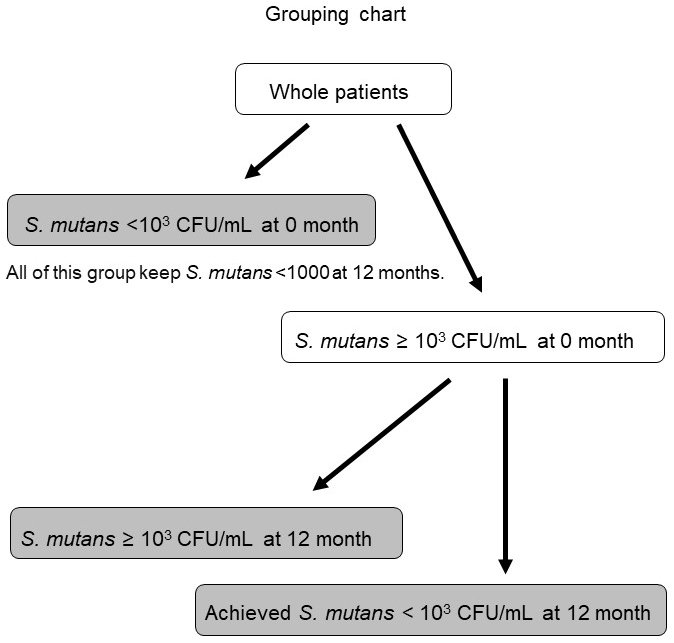
Grouping chart. Patients were divided into three groups: <10^3^ colony-forming units (CFU)/mL *S. mutans* at month 0, and those with < and ≥10^3^ CFU/mL *S. mutans* after 12 months.

The number of *S. mutans* decreased significantly (Friedman test, p < 0.001) after 12 months compared with month 0 (Bonferroni correction, p < 0.0167) (Figure 1a). Proteinuria also changed significantly over time (Friedman test, p < 0.01). Although there was no significant difference between 0 and 12 months, proteinuria decreased significantly from 6 to 12 months (Bonferroni correction, p < 0.0167) (Figure 1b). The relationship between the degree of decrease in the number of *S. mutans* after 12 months and proteinuria <0.3 g/gCr after 12 months remained significantly different in subsequent logistic regression analysis adjusted for age, sex, body mass index, systolic blood pressure, eGFR, proteinuria (g/gCr) at month 0, and renin-angiotensin system inhibitor use (p < 0.05) (Table 3). The degree of decrease in the number of *S. mutans* after 12 months was defined as the difference between the log-transformed number of *S. mutans* at 12 months and that at baseline (0 months). Patients were further divided into three groups: <10^3^ CFU/mL *S. mutans* at month 0, and those with < or ≥10^3^ CFU/mL after 12 months. As the group with <10^3^ CFU/mL *S. mutans* at month 0 showed no change in the number of *S. mutans* or proteinuria in the study period, this group was made independent (Figure 4). There was no significant difference in the rate of proteinuria <0.3 g/gCr at month 0 between the three groups (Table 4). However, the rate of proteinuria <0.3 g/gCr after 12 months was significantly higher in the <10^3^ CFU/mL group after 12 months compared with the other groups (Table 4).

### The relationship between decreased *S. mutans* abundance and decreased proteinuria in patients with IgAN and chronic glomerulonephritis

When assessing individual specimens, six patients achieved *S. mutans* <10^3^ CFU/mL after 12 months: three with IgA nephropathy or chronic glomerulonephritis and three with nephrosclerosis (Table 4). All three patients in the IgA nephropathy and chronic glomerulonephritis group also achieved proteinuria <0.3 g/gCr, whereas none of the patients with nephrosclerosis reached this proteinuria target. Additionally, the patient with diabetic nephropathy did not achieve <10^3^ CFU/mL *S. mutans* after 12 months. Because of this, patients with IgAN and chronic glomerulonephritis were selected for further analysis. There was no significant difference in the rate of proteinuria <0.3 g/gCr at month 0 between the groups (Figure 2a); however, the rate of proteinuria <0.3 g/gCr after 12 months was significantly higher in the group that had *S. mutans* <10^3^ CFU/mL after 12 months than in the other groups (Figure 2b).

## Discussion

To our knowledge, this is the first prospective study to explore a possible association between *S. mutans* oral cavity abundance and the degree of proteinuria in patients with CKD. While our findings are suggestive, the observational design does not allow us to establish causality, and residual confounding cannot be excluded. The ≥10^3^ CFU/mL *S. mutans* group had significantly higher instances of proteinuria than the <10^3^ CFU/mL group during the study period. The initial distribution of the number of *S. mutans* in the oral cavity was clearly divided into those with < and ≥10^3^ CFU/mL; therefore, it was reasonable to use this division to establish two groups. Oral cavity *S. mutans* <10^3^ CFU/mL is considered normal, while increased numbers are associated with increased dental caries ^(22), (23)^; because of this, it was also clinically reasonable to use this value as the cutoff line.

We also showed that oral care with chlorhexidine mouthwash may reduce proteinuria. As the number of *S. mutans* decreased significantly over time, so did proteinuria, with a delay. There was also a relationship between the decrease in the number of *S. mutans* and proteinuria <0.3 g/gCr after 12 months. Particularly in the regression analysis, the degree of reduction in *S. mutans* abundance, independent of proteinuria at month 0, affected the reduction in proteinuria after 12 months. Although various factors are considered to be associated with proteinuria, these results indicate that a higher number of *S. mutans* in the oral cavity may be one of these in patients with CKD.

Chlorhexidine mouthwashes are well-established oral care tools ^(6)^. This study provides preliminary evidence that they may reduce *S. mutans* counts and could also be linked to reductions in proteinuria. Recently, it has been reported that gargling with chlorhexidine may also improve diabetes ^(24)^. In the future, the possibility of improving systemic diseases through oral care will likely attract more attention.

Our results indicate that chlorhexidine mouthwash may be particularly effective in patients with CKD who have IgAN and chronic glomerulonephritis, and may be less effective in those with nephrosclerosis and diabetic nephropathy. In our study, only six individuals were able to reach* S. mutans* <10^3^ CFU/mL after 12 months. Among them, all with IgAN (n = 2) and chronic glomerulonephritis (n = 1) were able to achieve proteinuria <0.3 g/gCr after 12 months, while the three patients with nephrosclerosis could not. Additionally, a patient with diabetic nephropathy was not able to reduce *S. mutans* to <10^3^ CFU/mL, even after 12 months. These results may indicate that it is important to use mouthwash thoroughly to achieve *S. mutans* <10^3^ CFU/mL after 12 months, and that mouthwash use may be especially effective in patients with IgAN and chronic glomerulonephritis. Especially in IgAN and chronic glomerulonephritis, for which there are no specific treatments ^(25)^, the potential of oral care to improve proteinuria is of interest.

IgA nephropathy and chronic glomerulonephritis may be caused by a variety of factors, some of which may be related to oral bacteria. Recent clinical studies have demonstrated an association between IgAN and *cnm*-positive *S. mutans* in the oral cavity ^(10), (11), (12), (13), (14), (15), (17), (19)^. While previous studies have linked *cnm*-positive *S. mutans* to IgA nephropathy, the present study examined the overall* S. mutans* burden, and the efficacy of reducing *S. mutans* in IgA nephropathy has not yet been established. The *cnm* gene encodes a cell surface collagen-binding protein that can bind the extracellular matrix ^(26)^; this could be associated with various diseases such as cerebral hemorrhage ^(27), (28), (29)^, non-alcoholic steatohepatitis ^(30), (31)^, and inflammatory bowel disease ^(32)^, including IgAN ^(10), (11), (12), (13), (14), (15), (17), (19)^. One study revealed a significantly higher positivity rate of *cnm*-positive* S. mutans* in the oral cavity in patients with IgAN compared with healthy controls (32.1% vs. 14.0%) ^(10)^. Another study suggested an association between *cnm*-positive *S. mutans* in the oral cavity, dental caries, and urinary protein levels in patients with IgAN ^(11)^. In rodent models, the intravenous administration of *cnm*-positive* S. mutans* induced transient IgAN-like lesions ^(14)^. Severe dental caries induced by *cnm*-positive *S. mutans* were found to cause IgAN-like glomerulonephritis ^(15)^. This study found that not only *cnm*-positive *S. mutans,* but also an overall higher number of *S. mutans* in the oral cavity, may be associated with IgAN and other forms of glomerulonephritis. The possibility that a higher number of *S. mutans* may affect proteinuria is novel and very significant for future research. Our present findings are consistent with the new concept of an oral-kidney association ^(11)^.

This study had some limitations. First, it was a preliminary investigation and only indirectly demonstrated that a higher number of *S. mutans* in the oral cavity was associated with proteinuria; how this microbe contributes to proteinuria still needs to be determined. Additionally, few patients reached *S. mutans* <10^3^ CFU/mL or had significant decreases in proteinuria. It was also difficult to assess how thoroughly patients gargled. Although patients receiving immunosuppressive therapy were excluded, other factors that may affect *S. mutans* counts and proteinuria, including active infections, recent antibiotic use, severe oral disease, or poorly controlled diabetes, were not strictly controlled. Further verification of these results is needed by increasing the number of patients, ensuring proper adherence to mouthwash gargling, and adjusting for potential confounders. Future studies should include a two-group comparison between tap water and chlorhexidine. In addition, periodontal bacteria were not evaluated in this study, and the sample size was small, with all patients being of the same ethnicity and from a single center. Further studies with larger and more ethnically diverse populations across multiple facilities are warranted to confirm our findings.

Chlorhexidine also has certain risks. As there is evidence supporting the association between oral care with chlorhexidine and increased risk of mortality in patients on ventilator support, the Society for Healthcare Epidemiology, Infectious Diseases Society of America, and Association for Professionals in Infection Control and Epidemiology 2022 Guidelines recommend providing oral hygiene without chlorhexidine in their ventilator-associated pneumonia prevention bundles ^(33), (34), (35)^. However, these risks may be only for patients on ventilator support, and it is unclear whether they impact healthy subjects. The chlorhexidine used in these reports was also very concentrated (0.12%-2%) ^(33)^; as the gargle we used was a very low-concentration (0.00056%) and was spat out after gargling, the possibility of drug-related harm was very low. The safety of very low-concentration chlorhexidine gargles must still be confirmed. Overall, we found a potential association between oral *S. mutans* counts and proteinuria in patients with CKD; however, owing to the preliminary nature of this study and the limited sample size, we cannot conclude the effect of chlorhexidine mouthwash on proteinuria.

## Article Information

### Acknowledgments

We thank Ms. Airi Takeda for technical support with sampling. We thank Lisa Oberding, MSc, from Edanz (https://jp.edanz.com/ac) for editing a draft of this manuscript.

### Author Contributions

All authors contributed to the study conception.

Taro Misaki: Contributed to conception, data acquisition, statistical analyses, interpretation, and drafting of the manuscript.

Yuto Suehiro: Contributed to conception, data acquisition, and interpretation.

Shuhei Naka: Contributed to conception, data acquisition, and interpretation.

Daiki Matsuoka: Contributed to conception and data acquisition.

Kana Suehara: Contributed to conception and data acquisition.

Seigo Ito: Contributed to conception, statistical analyses, and interpretation of the manuscript.

Yasuyuki Nagasawa: Contributed to conception, interpretation, and critical revision of the manuscript.

Rena Okawa: Contributed to conception, data acquisition, and interpretation.

Ryota Nomura: Contributed to conception and interpretation.

Michiyo Matsumoto-Nakano: Contributed to conception, design, and interpretation.

Kazuhiko Nakano: Contributed to conception, design, interpretation, and critical revision of the manuscript.

All authors gave their final approval and agreed to be accountable for all aspects of the work.

### Conflicts of Interest

Kazuhiko Nakano has received research funding as the representative of the Joint Research Laboratory of Science for Oral and Systemic Connection, Graduate School of Dentistry, The University of Osaka. Yuto Suehiro has received salary support from research funds from the same laboratory. All other authors declare no competing interests.

### Ethical Statement

This study protocol fully adhered to the Declaration of Helsinki (64th WMA General Assembly, Fortaleza, Brazil, 2013). The protocol was approved by the Ethics Committee of Seirei Hamamatsu General Hospital (approval no. 3883), Osaka University Graduate School of Dentistry (approval no. R3-E23). All patients were informed of the study protocol and provided written informed consent before participating in the study.

### Data Availability Statement

The data are available from the corresponding author upon reasonable request.
